# The Development and Characterization of Novel Ionic Liquids Based on Mono- and Dicarboxylates with Meglumine for Drug Solubilizers and Skin Permeation Enhancers

**DOI:** 10.3390/pharmaceutics16030322

**Published:** 2024-02-26

**Authors:** Takayuki Furuishi, Sara Taguchi, Siran Wang, Kaori Fukuzawa, Etsuo Yonemochi

**Affiliations:** 1 Department of Physical Chemistry, School of Pharmacy and Pharmaceutical Sciences, Hoshi University, 2-4-41 Ebara, Shinagawa-ku, Tokyo 142-8501, Japanfukuzawa-k@phs.osaka-u.ac.jp (K.F.); 2 Graduate School of Pharmaceutical Sciences, Osaka University, 1-6 Yamadaoka, Suita, Osaka 565-0871, Japan

**Keywords:** ionic liquid, meglumine, solubility, transdermal, skin permeation enhancer

## Abstract

In this study, we synthesized a family of novel ionic liquids (ILs) with meglumine (MGM) as cations and tartaric acid (TA), azelaic acid (AA), geranic acid (GA), and capric acid (CPA) as anions, using pharmaceutical additives via simple acid–base neutralization reactions. The successful synthesis was validated by attenuated total reflection–Fourier transform infrared (ATR-FTIR) and powder X-ray diffraction (PXRD). Thermal analysis using differential scanning calorimetry confirmed the glass transition temperature of MGM-ILs to be within the range of −43.4 °C–−13.8 °C. We investigated the solubilization of 15 drugs with varying pKa and partition coefficient (log P) values using these ILs and performed a comparative analysis. Furthermore, we present MGM-IL as a new skin permeation enhancer for the drug model flurbiprofen (FRP). We confirmed that AA/MGM-IL improves the skin permeation of FRP through hairless mouse skin. Moreover, AA/MGM-IL enhanced drug skin permeability by affecting keratin rather than stratum corneum lipids, as confirmed by ATR-FTIR. To conclude, MGM-ILs exhibited potential as drug solubilizer and skin permeation enhancers of drugs.

## 1. Introduction

Ionic liquids (ILs) are ionic salts with a melting point below 100 °C, composed of relatively large-molecular-weight organic cations and organic/inorganic anions. It is well-known that a relatively strong electrostatic interaction force between cations and anions generates a liquid or viscous semi-solid state below 100 °C or even at ambient temperature. ILs have unique characteristics not found in other chemical substances, such as low vapor pressure, flame retardancy, high thermal and electrochemical stability, high electrical conductivity, and the ability to dissolve certain substances [1,2,3,4,5,6]. They are versatile solvents tailored to have specific physicochemical properties by selecting anion–cation combinations or introducing particular functional groups [3,4,5,6]. Due to their unique and useful properties, ILs can be confidently substituted for extremely volatile organic solvents as “green solvents” in various chemical processes such as synthesis and catalysis [7,8,9], extraction [10,11,12], and electrochemistry [13,14].

Transdermal drug administration offers several benefits over oral administration, including the avoidance of hepatic first-pass metabolism and intragastric pH changes, improved medical adherence, enhanced drug bioavailability, and direct delivery to the target site [15,16,17]. However, transdermal delivery of not only several poorly water-soluble drugs, but also highly water-soluble drugs, has proven challenging. Therefore, it is crucial to consider the issue of the water solubility of the candidate compound from the outset and improve its solubility.

A growing body of research is exploring the potential of ILs in applied pharmacology [18], given their ability to dissolve drugs with poor or no aqueous solubility. Developing drug delivery systems capable of enhancing both drug efficacy and bioavailability remains challenging in the pursuit of optimizing therapeutic outcomes. Various formulation strategies using nanocarriers such as lipid nanoparticles, polymeric nanoparticles, metal nanoparticles, graphene-based nanomaterials, microemulsion, and nanocrystals are continuously being explored to facilitate the targeted delivery of inadequately water-soluble therapeutic agents, but are also limited by use of organic solvents such as dichloromethane, dimethyl sulfoxide, acetonitrile, acetone, ethanol, hexane, and ethyl acetate during development, which can pose safety risks [19]. ILs are effective “solubilizers” of water-insoluble drugs, even those poorly soluble in organic solvents [20,21,22].

In addition, the use of ILs as penetration enhancers in transdermal drug delivery has been extensively documented [23,24,25,26]. For example, choline-based ILs enhance the skin permeability of hydrophobic and hydrophilic drugs [27]. This suggests that ILs hold great potential as additives in a wide range of dermal formulations.

Carbohydrates are emerging as promising starting materials for the next generation of ILs due to their reduced environmental impact during production and use [28,29]. N-methylglucamine, or meglumine (MGM), a valuable sugar-based IL [30,31,32,33,34,35,36,37,38,39,40], has demonstrated its potential for synthesis from D-glucose through reductive amination [41]. This method, which employs methylamine and H_2_ under high temperature and pressure conditions, provides a cost-effective and readily obtainable source of MGM, making it an attractive option for various applications. MGM is a recognized pharmaceutical excipient employed as a pH adjuster and solubilizer in solid oral and parenteral formulations, adhering to international regulatory guidelines. The remarkable versatility of MGM as a precursor for synthesizing novel ILs has led to the development of a diverse range of highly efficient solvents. These ILs exhibit exceptional performance in boron removal from water, metal-free catalysis for oxygen evolution, and ligand applications in high-performance liquid chromatography (HPLC) stationary phases, underscoring their potential as innovative functional materials [30,31,32,34,36,38,39,40]. Despite the well-established solubilizing properties of MGM and its widespread use as a pharmaceutical excipient, no studies have investigated the potential of MGM-based ILs in improving drug solubilization and skin drug permeation.

**Figure 1:**
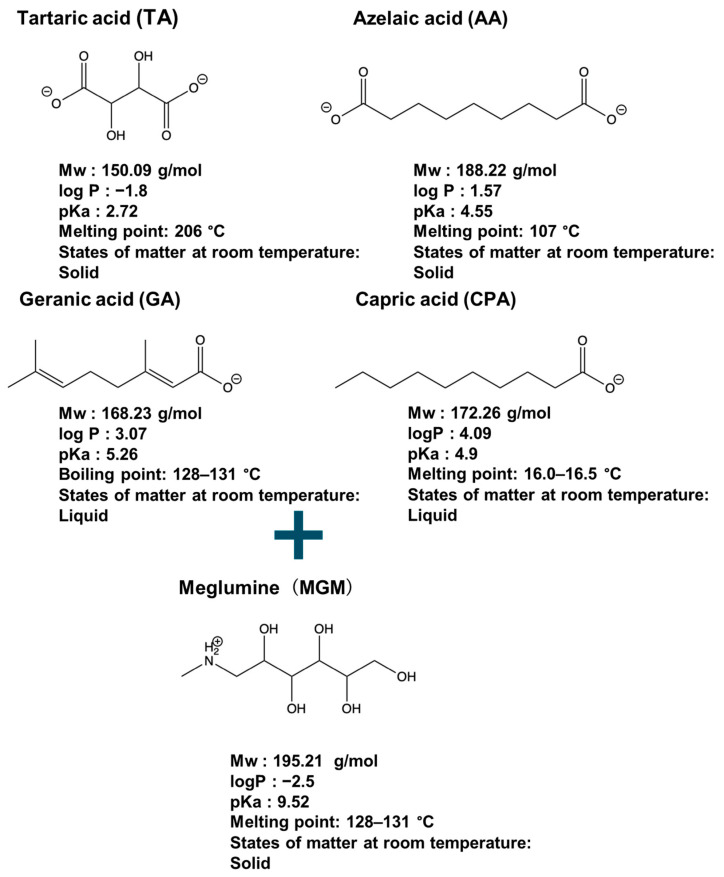
Chemical structures and characterizations of organic acids and MGM.

In this study, four novel ILs with MGM as cations and tartaric acid (TA) [42], azelaic acid (AA) [43], geranic acid (GA) [44], and capric acid (CPA) [45] as anions, which are candidates of anions for ILs, were synthesized using pharmaceutical additives via simple acid–base neutralization reactions (Figure 1). The synthesized ILs were characterized by attenuated total reflection–Fourier transform infrared (ATR-FTIR) spectroscopy, differential scanning calorimetry (DSC) analysis, and powder X-ray diffraction (PXRD). The solubilization of a range of drugs with diverse physical properties (Table 1) was investigated using these ILs, followed by a comparative assessment of their solubilization efficacy. Additionally, skin permeation studies were conducted to evaluate the permeation-enhancing potential of ILs. No previous reports have explored the drug skin permeation profiles of these anions in combination with MGM. Moreover, we examined the influence of ILs on the stratum corneum (SC) lipids, as evaluated by ATR-FTIR measurements, to shed light on the mechanisms behind the improved drug skin permeation.

## 2. Materials and Methods

### 2.1. Materials

TA, Captopril (CPP), and Disopyramide (DPA) were obtained from FUJIFILM Wako Pure Chemical Corporation (Osaka, Japan). AA, allopurinol (ALP), ofloxacin (OFLX), isosorbide mononitrate (ISMN), atenolol (ATL), minoxidil (MXD), carbamazepine (CBZ), curcumin (CCM), flurbiprofen (FRP), carvedilol, (CVD), and coenzyme Q10 (CoQ10) were procured from Tokyo Chemical Industry (Tokyo, Japan). GA, meglumine (MGM), ferulic acid (FA), ethenzamide (ETZ), and white petrolatum were purchased from Sigma-Aldrich (Tokyo, Japan), Nippon Boehringer Ingelheim (Tokyo, Japan), Combi-Blocks, Inc. (San Diego, CA, USA), Junsei Pharmaceutical Industry (Tokyo, Japan), and Yoshida Pharmaceutical Co., Ltd. (Tokyo, Japan), respectively. Capric acid (CA) and Rutin were obtained from Nacalai Tesque (Kyoto, Japan). The rest of the reagents were of analytical grade, readily available commercially, and employed without further purification.

### 2.2. Preparation of ILs between Organic Acids and MGM

Appropriate corresponding molar amounts of organic acid and MGM were mixed in the screw tube using a vortex mixer, and water and ethanol as the solvent were added. After having been completely dissolved by ultrasound, the resulting solution was evaporated using a thermostat at 65 °C and then dried under reduced pressure at 40 °C for 24 h.

### 2.3. Characterization of ILs between Organic Acids and MGM

#### 2.3.1. Attenuated Total Reflection (ATR)–Fourier Transform Infrared (ATR-FTIR) Measurements

Infrared spectra were determined using the ATR method on an FTIR-4200 spectrometer (Jasco Co., Tokyo, Japan) equipped with an ATR unit (ATR PRO 670H-S, Jasco) and an internal reflection element (i.e., a 45° trapezoid diamond with entrance and exit faces). The detector used was a mercury cadmium telluride detector (MCT-4000M, Jasco). The sample was scanned 64 times, and the spectra were acquired from 4000 to 400 cm^−1^ with a resolution of 4 cm^−1^ at 25 °C.

#### 2.3.2. DSC Measurements

DSC measurements were performed using a Thermoplus EVO DSC 8230 (Rigaku Corporataion, Tokyo, Japan) instrument equipped with a gas selector (Rigaku) and a liquid nitrogen (LN_2_) controller. Approximately 3–5 mg of the sample was weighed into an aluminum pan sealed with an aluminum lid. An empty pan with the same characteristics was used as a control. The samples were subject to a heat–cool–heat cycle. Under a nitrogen purge of 100 mL min^−1^, the specimen was heated to 50 °C at 5 °C min^−1^ and held isothermally for 10 min to erase the thermal history. Next, the specimen was cooled to −70 °C at a rate of 5 °C min^−1^, held isothermally for 5 min, and reheated to 100 °C. LN_2_ was used for cooling.

### 2.4. Solubility Capability Examination of Drug-In ILs between Organic Acids and MGM

Appropriate equimolar amounts of MGM and organic acid were mixed in the screw tube using a vortex mixer. Next, 1, 10, 20… *w*/*w*% of each drug and water and ethanol as the solvent was added to the MGM and organic acid solution, and then the preparation was sonicated to be completely dissolved. The resulting solution was evaporated using a thermostat at 65 °C and then vacuum-dried at 40 °C for 24 h. PXRD, described in Section 2.3.1, was performed to confirm the solubility capability of the drug-in IL.

### 2.5. Skin Permeation Studies

Hairless mouse skin (Labo Skin, HOS: HR-1 Male, 7 weeks, Hoshino Laboratory Animals, Inc., Ibaraki, Japan) was resected on a Franz-type diffusion cell (Osawa Shokai Co. Ltd., Tokyo, Japan). For the skin permeation test, we prepared 200 mg of 1 *w*/*w*% FRP-in-IL (containing 2 mg of the drug) and conducted the test in a Franz diffusion cell (Osawa Shokai Co., Ltd.) for 24 h at 32 °C through the mice skins. We positioned the skin between the donor and receptor cell, ensuring that the adjacent dermis was in contact with the receiver section. Subsequently, we added 200 mg of prepared FRP-in-IL and 7.0 mL of phosphate-buffered saline (PBS, pH = 7.4) to the donor and receptor cells, respectively, with an accessible diffusion zone of 1.13 cm^2^. The receptor solution was maintained at 32 °C and stirred at 300 rpm during the trial. Receptor solution samples (0.5 mL) were collected at encoded intervals (0, 1, 2, 3, 4, 5, 6, 7, 8, 22, and 24 h) and immediately replaced with an equal volume of fresh receptor medium. The sample FRP concentration was then determined via HPLC analysis. White petrolatum was used as a control base.

### 2.6. Analytical Method

The HPLC system consisted of a PU-plus intelligent HPLC pump, a UV-intelligent UV/VIS detector, a CO-2060 plus intelligent column oven, an AS-2055 plus intelligent sampler, and a ChromNAV chromatography data system, ver. 1.08 (all equipment provided by Jasco). The analytical column was an Inertsil ODS-3 column (150 × 4.6 mm i.d., particle size: 5 μm) provided by GL-Sciences (Tokyo, Japan), operated at 40 °C. The mobile phase was a mixture of PBS (pH 7.0) and acetonitrile (80:20, *v*/*v*) at a flow rate of 0.8 mL/min. The injection volume was 20 μL, and the column eluate was monitored at a wavelength of 247 nm.

### 2.7. ATR-FTIR Assessment of the SC Samples

The MGM-based IL was prepared in a glass Petri plate using the method described in Section 2.2. The SC sheet was gently peeled off, immersed in the glass Petri plate, and incubated at 32 °C for 24 h. The samples were then rinsed with PBS, with the PBS-treated SC sheet as the control. We inspected the SC samples using ATR-FTIR, acquiring 256 scans from 400 to 4000 cm^−1^ at a resolution of 2 cm^−1^.

### 2.8. Statical Analysis

We conducted all the experiments in triplicate and analyzed the results using a one-way analysis of variance followed by modified Fisher’s least-squares difference post hoc testing. Statistical significance was set at *p* < 0.05.

## 3. Results and Discussion

### 3.1. Preparation of MGM-ILs

**Figure 2:**
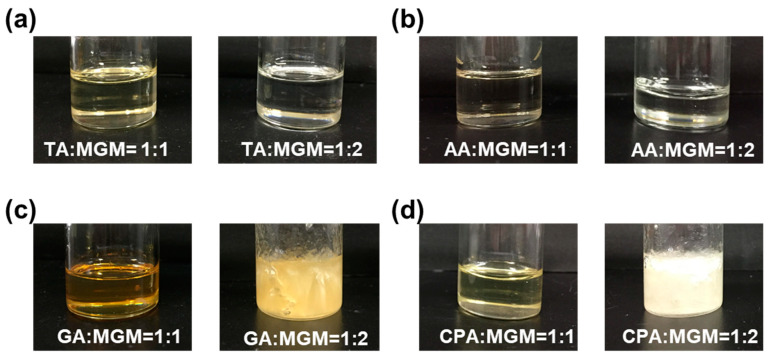
Visual characterization of the synthetized ILs based on MGM with organic acids at a molar ratio of organic acid to MGM of 1:1 and 1:2 (TA: MGM, (**a**)), (AA: MGM, (**b**)), (GA: MGM, (**c**)), and (CPA: MGM, (**d**)).

ILs can be synthesized simply and conveniently via ultrasound-assisted neutralization reactions, owing to the several advantages of ultrasound, including improved reaction rates, reduced reaction times, and the avoidance of harsh experimental conditions. The synthesis of the ILs involved the utilization of TA, AA, GA, CA, and MGM, commonly employed as pharmaceutical additives. The obtained complexes presented as colorless or light-yellow transparent gels, except for GA-MGM 1:2 and CPA-MGM 1:2, which exhibited a turbid appearance (Figure 2).

### 3.2. Characterization of MGM-ILs

**Figure 3:**
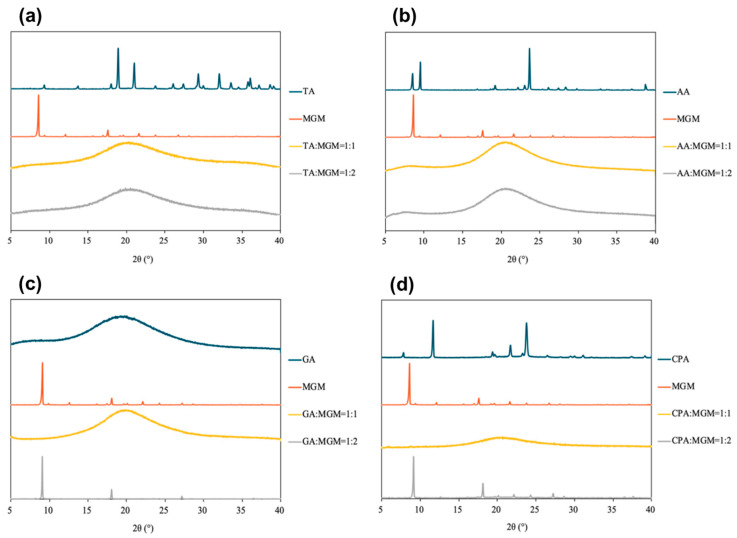
PXRD patterns of ILs (TA: MGM, (**a**)), (AA: MGM, (**b**)), (GA: MGM, (**c**)), and (CPA: MGM, (**d**)).

The PXRD patterns of the MGM-based ILs are shown in Figure 3. All the transparent gel complexes exhibited an amorphous state, indicating a lack of long-range order in their molecular arrangement. In contrast, the PXRD patterns of GA-MGM 1:2 and CPA-MGM 1:2 showed distinct peaks corresponding to MGM-derived crystals, suggesting the precipitation of excess MGM. When considered together with Figure 1, the PXRD data suggest that GA-MGM 1:2 and CPA-MGM 1:2 cannot form ILs.

**Figure 4:**
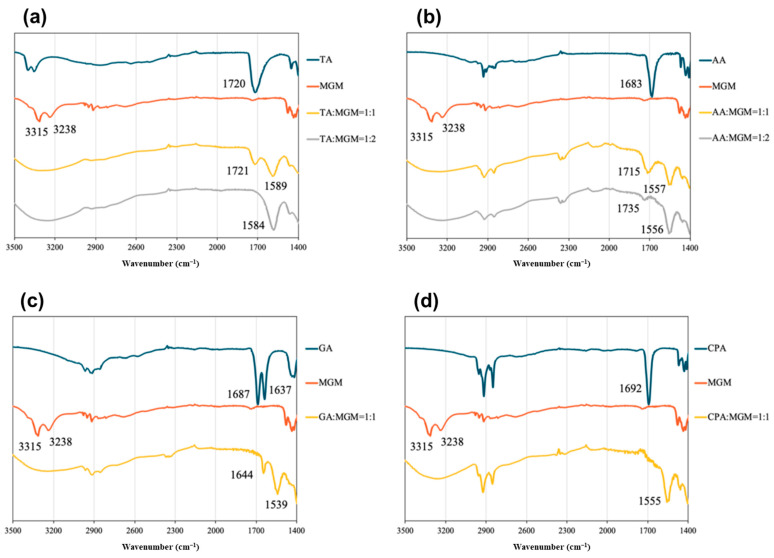
ATR-FTIR spectra of (**a**) TA/MGM, (**b**) AA/MGM, (**c**) GA/MGM, and (**d**) CPA/MGM systems.

Given that ATR-FTIR spectra provide insights into the functional group state of molecules, FTIR spectroscopy was utilized to detect the interaction between the IL components and validate salt formation [46]. Figure 4 shows the ATR-FTIR spectra of the ILs, in which the characteristic absorption peaks of the C=O vibration of the COOH group were observed at 1720 cm^−1^ in TA, 1683 cm^−1^ in AA, 1687 cm^−1^ in GA, and 1692 cm^−1^ in CPA. However, the asymmetric stretching vibration of the COO^−^ group appeared at 1584 cm^−1^ in TA-MGM 1:2, 1556 cm^−1^ in AA-MGM 1:2, 1539 cm^−1^ in GA-MGM 1:1, and 1555 cm^−1^ in CPA-MGM 1:1, respectively [47]. The dicarboxylic acids TA and AA, at a 1:1 molar ratio, exhibited two absorption peaks at 1721 and 1589 cm^−1^, and 1715 and 1557 cm^−1^, respectively, which were attributed to the C=O vibrations of the COOH and COO^−^ groups. Moreover, the peak attributed to the C=C bond shifted from 1644 cm^−1^ to 1637 cm^−1^ in GA-MGM 1:1. Moreover, the sharp amine-derived peaks at 3315 and 3238 cm^−1^ in MGM disappeared and were replaced by a broad absorption band (3000–3500 cm^−1^) in all ILs. The disappearance of the stretching vibration of the free carboxylic acid and amine groups indicates compound ionization and salt formation.

**Figure 5:**
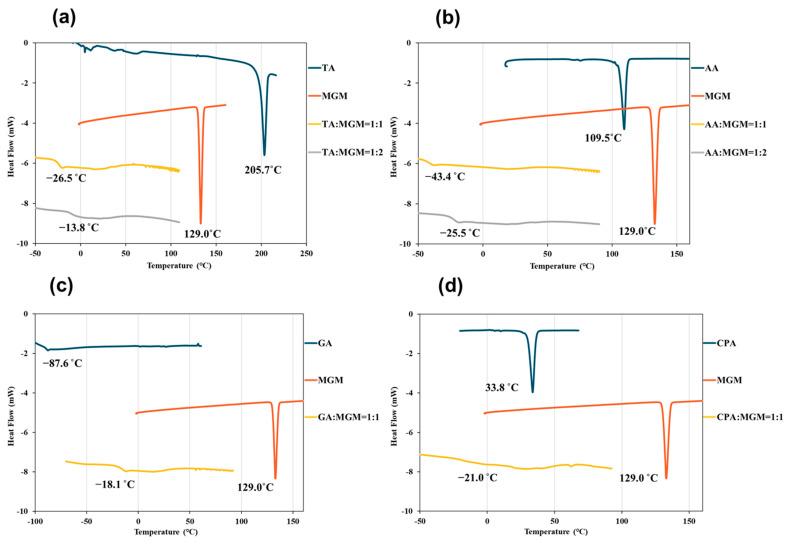

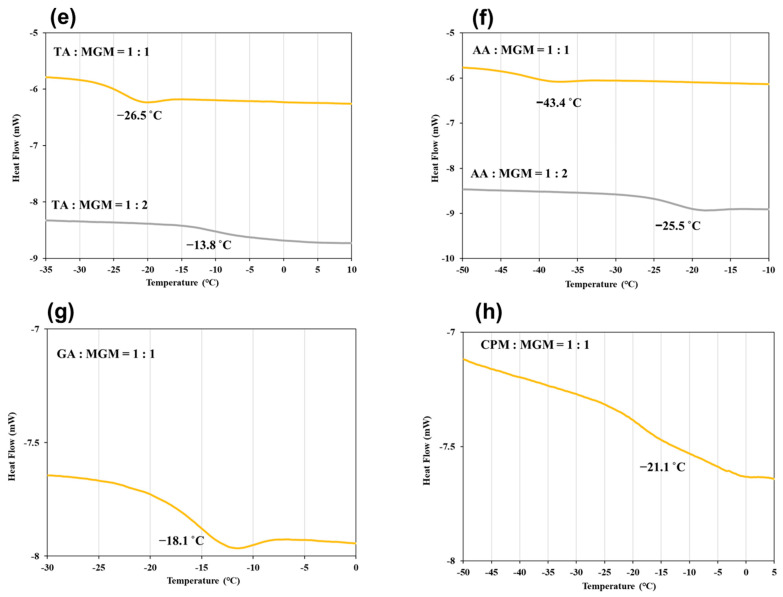
DSC profiles of TA/MGM (**a**), AA/MGM (**b**), GA/MGM (**c**), and CPA/MGM (**d**), and *T*g points of TA-MGM = 1:1 and 1:2 (**e**), AA-MGM = 1:1 and 1:2 (**f**), GA-MGM = 1:1 (**g**), and CPM-MGM = 1:1 (**h**) systems.

Further characterization of the MGM-based ILs was conducted using DSC measurements (Figure 5). The DSC thermograms revealed an endothermic peak for MGM at 129 °C, while those for TA, AA, GA, and CA were observed at 205.7 °C, 109.5 °C, −87.6 °C, and 33.8 °C, respectively. While no distinct melting points were observed within the temperature range scanned for these MGM-based ILs, the DSC thermograms exhibited changes in heat capacity, indicating the presence of *T*g that differs from the melting point of either MGM or the individual organic acids. Figure 5 shows that the *T*g values of the ILs ranged from −43.4 °C to −13.8 °C, depending on the anion structure and the ratio of MGM to acids [1,48]. Comparing the *T*g of TA-MGM 1:1 and 1:2, TA-MGM 1:2 shows a higher *T*g value than TA-MGM 1:1, the same as the AA-MGM system. This indicates that TA-MG 1:2 and AA-MGM 1:2 form stable ILs. Combined with the results of visual characterization (Figure 2), PXRD profiles (Figure 3) and ATR-FTIR spectra (Figure 4), it is suggested that TA-MGM and AA-MGM ILs are formed at a molar ratio of 1:2, whereas GA-MGM and CPA-MGM formed 1:1 ILs.

### 3.3. MGM-ILs’ Solubility

**Figure 6:**
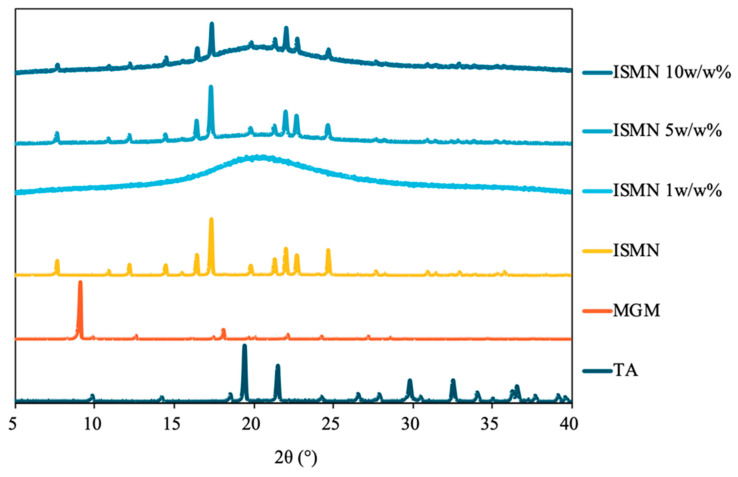
PXRD pattern of drug (ISMN) in IL (TA-MGM 1:2).

The solubilization power of ILs is attributed to a range of interactions, such as ionic, van der Waals, π–π, and hydrogen bonding. This suggests that ILs could have a greater solubilization power than traditional organic solvents [49]. To determine the solubility capability of the ILs, drug-in-IL complexes were prepared. Subsequently, these complexes were analyzed via PXRD to confirm dissolution. PXRD is a technique used to validate the crystalline structure of a drug or excipients in a preparation. The absence of a drug’s crystal form in PXRD indicates the complete solubilization of the drug in the IL. For 5% and 10% ISMN in TA-MGM 1:2, the presence of drug-derived crystal peaks suggests that the drug cannot be completely dissolved (Figure 6).

**Figure 7:**
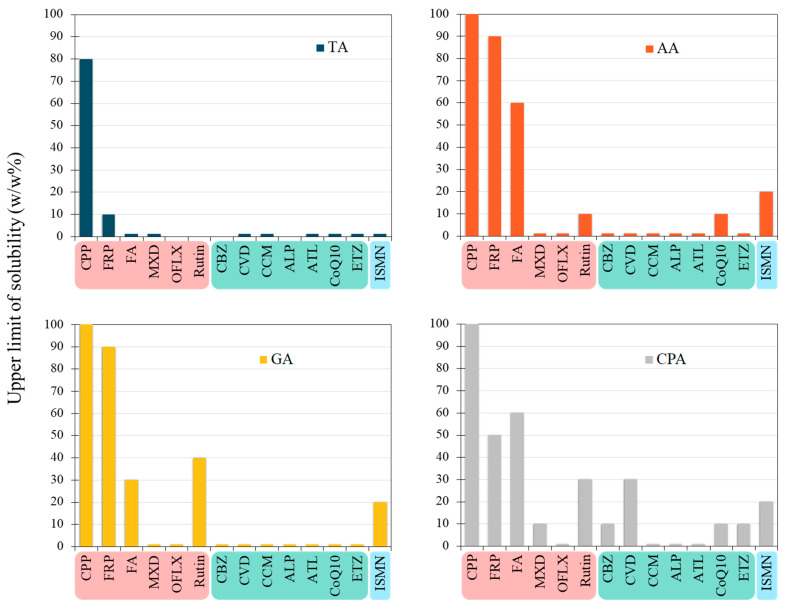
The correlation between upper limit of dissolution and each drug in ILs (TA/MGM; AA/MGM; GA/MGM; CPA/MGM).

The upper limit of drug dissolution in the ILs is shown in Figure 7, with the pKa value increasing from left to right. In TA-MGM 1:2, CPP and FRP could dissolve up to 80% and 10%, respectively. In AA-MGM 1:2, CPP, FRP, FA, ISMN, Rutin, and Coenzyme Q10 dissolved at concentrations greater than 10%. In GA-MGM 1:1, CPP, FRP, FA, ISMN, and Rutin dissolved at a concentration of up to 20% or more. In CPA-MGM 1:1, all drugs except OFLX, CCM, ALP, and ATL dissolved at concentrations of 10% or higher. Acidic compounds with lower pKa values appeared to have greater solubility in these four ILs than other compounds. The MXD molecule, a weakly acidic drug with a pKa of 4.61, surprisingly exhibited poor solubility in ILs despite containing nitrogen heterocycles and amino groups. OFLX was soluble in both acids and bases, likely due to its steric hindrance and relatively rigid structure, which may hinder its dissolution in ILs. Rutin and ISMN also exhibited greater solubility than other basic drugs, likely due to the absence of amines, the presence of hydroxyl groups in their structure, and their relatively low partition coefficient (log P) values. DPA, which tends to form an amorphous state, exhibited 100% solubility in all ILs.

MGM-ILs based on AA, GA, and CPA with longer side chains and higher lipophilicity exhibit an excellent solubility capability for a wide range of drugs with diverse properties. Specifically, CPA-MGM 1:1 may have surfactant-like properties with an appropriate HLB value due to the amphiphilic nature, resulting in good solubilization [50,51,52]. Additionally, Raihan Chowdhury et al. reported that the density and viscosity of ILs increased with the amino acid chain length, resulting in increased paclitaxel solubility [53]. This suggests that the alkyl chain length of the ions in ILs can significantly improve drug solubility. Consequently, the impact of both the structures of ILs and drugs on the improvement of drug solubility is complex [54].

### 3.4. In Vitro Skin Permeation Test

The transdermal permeation of drug-in-IL complexes was investigated using Franz diffusion cells with hairless mouse skin. FRP, a lipophilic drug previously studied in transdermal systems, was used as the model drug [55,56]. To improve FRP percutaneous absorption through ionic liquidation, we have previously reported that the drug–drug IL of FRP and lidocaine, a local anesthetic, solves the problem of skin permeation [57]. Although some progress has been made, further research is needed to optimize the use of ILs as skin permeation enhancers for FRP transdermal delivery.

**Figure 8:**
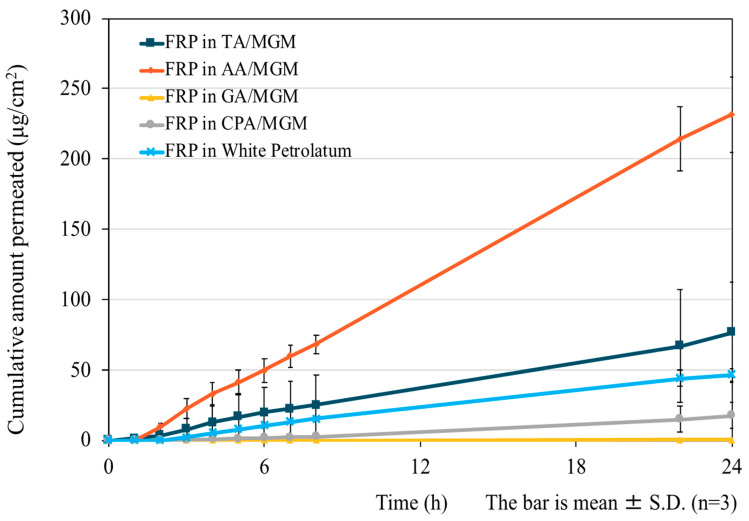
Skin permeation profiles of 1%FRP in ILs and white petrolatum.

Figure 8 shows the results of the skin permeation test. For GA-MGM 1:1 and CPA-MGM 1:1, which have good solubility for FRP, the fluxes were low, at 0.86 and 0.035 μg/cm^2^/h, respectively. This may be due to the entrapment of drug molecules in the strong affinity matrix [58]. In AA-MGM 1:2 and TA-MGM 1:2, the fluxes were 8.78 and 3.53 μg/cm^2^/h, respectively, approximately 3.2-fold and 1.3-fold higher than the control (flux: 2.73 μg/cm^2^/h). The permeation enhancement effects of TA-MGM 1:2 and AA-MGM 1:2 may be attributed to the presence of MGM. Moreover, AA is a medium–long-chain dibasic acid well-known for decreasing keratin production. Transdermal absorption enhancers typically decrease skin barrier properties by disrupting regular and compact corneocyte arrangement. The mechanism of IL penetration may involve disrupting the orderly structure of the SC lipids. However, the precise effects of ILs on the SC lipid structure remain unclear, and further investigation using ATR-FTIR is needed.

### 3.5. ATR-FTIR Assessment of the MGM-IL-Treated SC

In recent years, ILs have emerged as a promising class of permeation enhancers for transdermal drug delivery. ILs have been shown to enhance transdermal drug delivery systems through both transcellular and paracellular pathways by disrupting cellular integrity, fluidizing the SC lipid matrix, forming diffusional pathways, and extracting lipid components from the SC [59]. Usually, transdermal absorption enhancers typically decrease skin barrier properties by disrupting regular and compact corneocyte arrangement [60].

**Figure 9:**
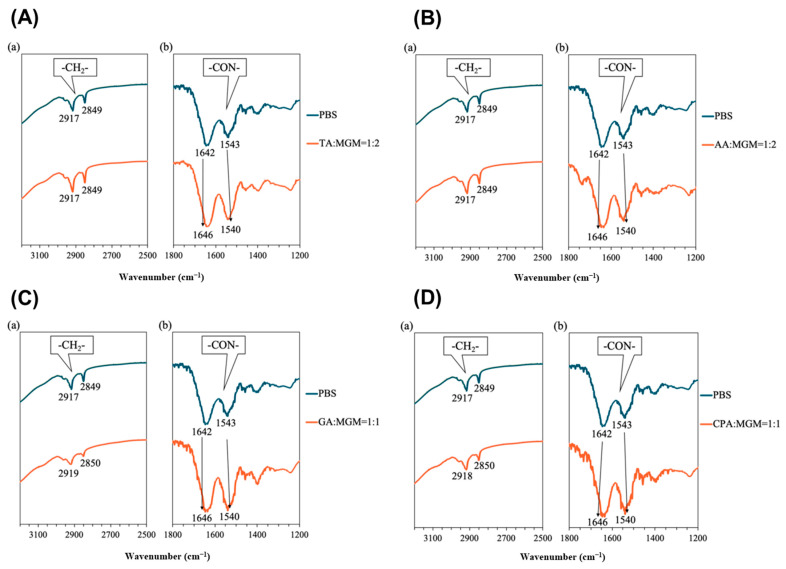
FTIR spectra for hairless mouse skin treated with IL (TA:MGM = 1:2, (**A**)), (AA:MGM = 1:2, (**B**)), (GA:MGM = 1:2, (**C**)), and (GA:MGM = 1:2, (**D**)).

ATR-FTIR is a widely used technique to gain insights into the molecular organization of the lipid matrix in the SC [61,62,63]. The peaks obtained around 2920 and 2850 cm^−1^ correspond to the asymmetric and symmetric stretching modes of the terminal methylene groups in the lipids, respectively, providing valuable information on the internal structure of the lipid bilayer. As show in Figure 9, the reference specimen exhibited lipid absorption peaks at 2917 cm^−1^ (C–H asymmetric vibration) and 2849 cm^−1^ (C–H symmetric vibration). And the characteristic absorption peaks of keratin were observed at 1642 cm^−1^ and 1543 cm^−1^ (NH–C=O vibration) [64].

No alterations were observed in the peak corresponding to the lipid alkyl chain in the skin treated with TA/MGM-IL (Figure 9A(a)) and AA/MGM-IL (Figure 9B(a)). A series of characteristic bands associated with keratinocyte structures were detected in the range of 1800–600 cm^−1^. The overlapping of multiple bands in this region (1700–1500 cm^−1^) highlights the intricate nature of the spectral pattern, reflecting the presence of numerous protein-derived bands (amides I–VII). This region encompasses the most prominent structural features in the ATR-FTIR spectrum of skin, arising from the amide I and amide II bands [65]. The amide I and amide II bands, in particular, are of paramount importance for analysis due to their sensitivity to changes in protein structure. These bands are directly correlated with the secondary structure of proteins in keratinocytes [66,67].

In contrast, the amide-derived peaks of skin proteins exhibited a shift of approximately 4 cm^−1^ upon treatment with TA/MGM-IL and AA/MGM-IL compared to the PBS treatment (Figure 9A(b),B(b)), implying an interaction between these ILs and skin proteins. The data suggest that TA/MGM-IL and AA/MGM-IL promote drug skin permeation by affecting keratin rather than SC lipids. Conversely, GA/MGM-IL and CPA/MGM-IL caused redshifts in the absorption peaks of both SC lipids and keratin (Figure 9C,D). The CH stretching vibrations shifted to 2919 cm^−1^ (asymmetric) and 2850 cm^−1^ (symmetric), while the NH–C=O vibrations of keratin exhibited shifts to 1646 cm^−1^ and 1540 cm^−1^, respectively. These alterations are directly linked to the molecular arrangements within the SC.

## 4. Conclusions

The formation of MGM-ILs occurs primarily through ionic interactions and hydrogen bonds rather than covalent bonds [68]. The observed changes in the absorption bands corresponding to –NH and C=O groups in the ATR-FTIR spectra suggest alterations in the systemic structures, thereby reflecting modifications in intermolecular forces. Notably, the wavenumber shifts of the C=O bond around 1550 cm^−1^ in MGM-ILs indicated asymmetric stretching vibrations of carboxylic salts. Compared to inorganic salts, the relatively weak ionic interactions among organic compounds in MGM-ILs cause the lack of an initial ordered arrangement of molecules and disrupt the overall crystallinity of the structure, resulting in *T*g ranging from −43.4 °C to −13.8 °C.

The screening experiment revealed that MGM-ILs can solubilize a wide range of drug molecules with varying pKa and log *p* values, making them promising candidates as drug carriers. Among the tested MGM-ILs, AA/MGM-IL exhibited a greater penetration effect when utilized as a semi-solid formulation base. Based on the available evidence, it is plausible that AA/MGM-IL has a minimal impact on the lipids present within the stratum corneum, but rather promotes drug penetration by modulating the keratin structure, as suggested by ATR-FTIR data [26].

## Figures and Tables

**Table 1:** Chemical structures and characterizations of drugs used in this study.

Drug	Structure	Log P	pKa	m.p. (°C)	Mw (g/mol)	CAS Number
Rutin	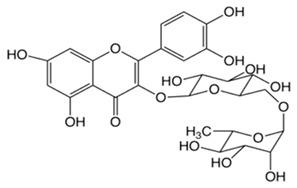	−1.33	6.43	214–215	611	207671-50-9
Ofloxacin (OFLX)	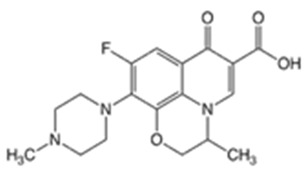	−0.48	pKa1 = 5.97 pKa2 = 9.28	270–275	361	82419-36-1
Captopril (CPP)	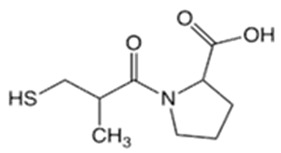	0.34	3.64	105–110	217	62571-86-2
Minoxidil (MXD)	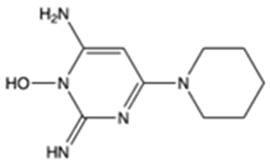	1.24	4.61	272–274	209	38304-91-5
Ferulic acid (FA)	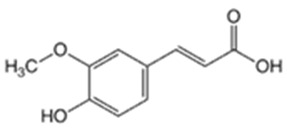	1.51	4.58	173–176	194	1135-24-6
Flurbiprofen (FRP)	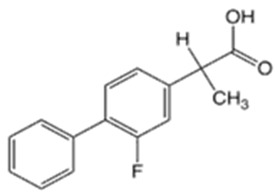	4.16	3.78	110–112	244	5104-49-4
Isosorbide mononitrate (ISMN)	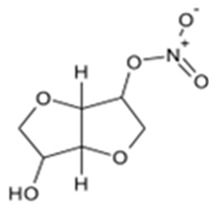	−0.15	--	88–93	191	16051-77-7
Allopurinol (ALP)	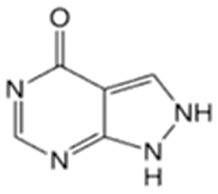	−1.80	9.34	>300	136	315-30-0
Atenolol (ATL)	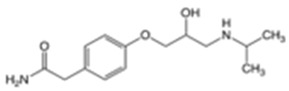	0.16	9.6	152–156	266	29122-68-7
Ethenzamide (ETZ)	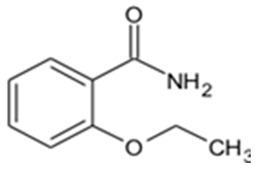	1.02	13.7	270–273	165	938-73-8
Carbamazepine (CBZ)	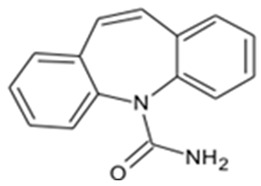	2.54	7.00	189–193	236	298-46-4
Disopyramide (DPA)	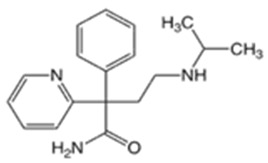	2.58	8.36	85–87	339	3737-09-5
Curcumin (CCM)	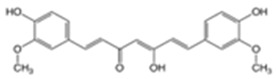	3.29	8.09	179–185	368	458-37-7
Carvedilol (CVD)	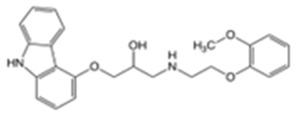	4.19	7.8	114–119	406	72956-09-3
Coenzyme Q10 (CoQ10)	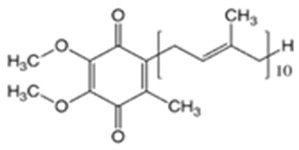	9.94	10.9	47–52	863	303-98-0

Log P: Log (partition coefficient). pKa: −Log (the acid dissociation constant). m.p.: melting point. Mw: molecular weight.

## Data Availability

Data will be made available on request.
